# Improving Quality Management of ‘Ready‐To‐Eat’ Pomegranate Arils Using *Aloe Ferox* ‐Based Edible Coating Enriched With Encapsulated Raspberry Pomace

**DOI:** 10.1002/fsn3.70636

**Published:** 2025-07-14

**Authors:** N. P. Mbonambi, F. Seke, S. Mwelase, O. A. Fawole

**Affiliations:** ^1^ Postharvest and Agroprocessing Research Centre, Department of Botany and Plant Biotechnology University of Johannesburg Johannesburg South Africa; ^2^ South African Research Chairs Initiative in Sustainable Preservation and Agroprocessing Research University of Johannesburg Auckland Park South Africa; ^3^ Department of Horticulture Faculty of Applied Sciences, Durban University of Technology Durban South Africa

**Keywords:** cold storage, functional coatings, minimally processed fruits, postharvest treatment, quality, valorisation

## Abstract

The labour‐intensive process of extracting arils from the pomegranate fruit has given rise to the demand for ‘ready‐to‐eat’ pomegranate arils due to their aesthetic appeal and convenience in consumption. However, the arils are prone to rapid quality deterioration due to various factors such as microbial contamination and moisture loss; therefore, there is a need for techniques to preserve their quality. This study, thus, evaluated the use of an *Aloe ferox* (AF)‐based edible coating enriched with encapsulated raspberry pomace powder (ERPP) to maintain quality and extend the shelf life of pomegranate arils (cv. Wonderful) during cold storage. Treatments included control (distilled water), AF (3%), AF (3%) + ERPP (0.3%), and AF (3%) + ERPP (0.6%). The findings demonstrated that all coating treatments showed improved effectiveness in minimizing weight loss and respiration rate while preserving texture, TSS, and TPC compared to the control treatment. Among the coating treatments, the combination of AF with 0.3% ERPP showed superior efficacy in preserving the quality of pomegranate arils compared to AF alone or AF with 0.6% ERPP. This eco‐friendly coating shows potential for commercial application and warrants further investigation.

## Graphical abstract

This study evaluated an *Aloe ferox* ‐based edible coating enriched with encapsulated raspberry pomace powder (ERPP) to preserve the quality and extend the shelf life of ready‐to‐eat pomegranate arils during cold storage. The AF + 0.3% ERPP treatment was most effective in reducing weight loss and respiration rate while maintaining texture, TSS, and TPC, demonstrating its potential for commercial application as a natural preservation solution.

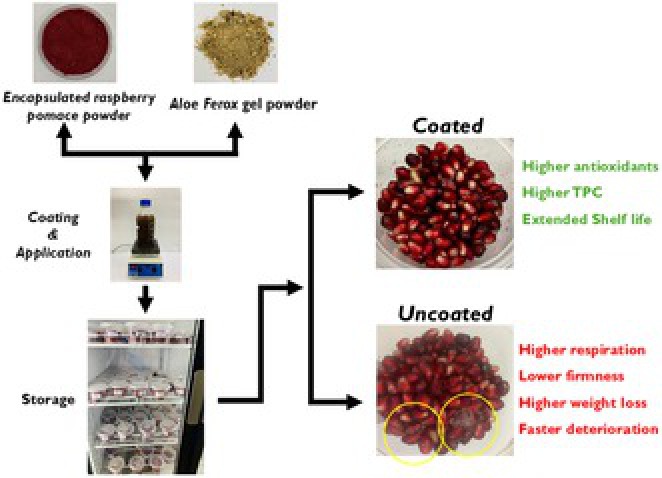

## 1 Introduction

Pomegranate ( *Punica granatum* L.) is a nutritious fruit rich in phytochemicals, including vitamins, minerals, anthocyanins, and phenolic compounds (Fawole et al. 2012, 2020). Recently, there has been a significant increase in the global commercial farming of pomegranates, driven by their recognized health benefits, including high antioxidant, anti‐mutagenic, and antihypertensive properties (Fawole and Opara 2013a; Mwelase et al. 2023). However, pomegranates are susceptible to rind infections during storage, impacting their appearance (Mwelase et al. 2023). Moreover, intake is frequently constrained by the challenge of extricating the arils from the fruit (Özdemir and Gökmen 2017). To address this issue and encourage increased fruit intake, the industry has redirected its efforts towards producing fresh‐cut or minimally processed items, such as ready‐to‐eat arils, which offer convenience and preserved quality (Rokalla et al. 2022). However, the pomegranate industry faces unique challenges in meeting consumer demand for ‘ready‐to‐eat’ arils. Pomagranate arils have a short shelf life due to microbial growth and accelerated quality degradation (Singla et al. 2022). This degradation is mainly caused by active metabolic processes, including endogenous enzymatic activity and increased respiration rates, which are higher in minimally processed products (Moradinezhad et al. 2020). Therefore, to meet consumer demands for fresh, healthy, and nutritious food, the food sector must implement new processing and creative food packaging technologies that ensure product quality and safety while maintaining consumer convenience (Giannoglou et al. 2020).

Technologies such as modified atmosphere (MA) packaging have been shown to delay quality losses and extend the shelf life of minimally processed pomegranate arils (Ayhan and Eştürk 2009). However, research indicates that arils packaged under MA conditions typically have a shelf life of only 10 days (Moradinezhad et al. 2020), which is further reduced to 7 days when considering sensory attributes (Caleb et al. 2013). These limitations highlight the need for innovative solutions to extend shelf life further while preserving quality (Martínez‐Romero et al. 2013). As a result, edible coatings are considered alternatives for reducing microbial growth and delaying quality degradation in pomegranate arils (Li et al. 2021; Ali et al. 2025). Edible coatings offer a partial barrier to moisture, oxygen, and carbon dioxide, helping to maintain freshness and improve mechanical handling (Kocira et al. 2021; Heydari et al. 2020).

Research has primarily focused on using traditional polysaccharides‐based edible coatings such as chitosan (Zarbakhsh et al. 2020), *Aloe vera* (Martínez‐Romero et al. 2013; Kaur et al. 2024), cellulose (Kawhena et al. 2020), and starch (Oz and Ulukanli 2012) to preserve quality pomegranate arils. Chitosan‐based polysaccharide coatings effectively inhibit microbial growth and maintain postharvest quality, but their non‐vegetarian origin limits their acceptability among vegetarians (Singla et al. 2022). As an alternative, *Aloe ferox* gel has emerged as a promising natural coating due to its unique biochemical composition and polysaccharides, which contribute to its effectiveness in food preservation and quality enhancement (Setsiba and Fawole 2022).

*Aloe ferox* , the second most utilized *Aloe* species after *Aloe vera* , is increasingly popular due to its antioxidant, anti‐inflammatory, antibacterial, anticancer, antimalarial, and anthelmintic properties (Egbuna et al. 2020). Approved by the FDA as a natural food additive (FDA 2002), *Aloe ferox* aligns with consumer demands for sustainable, biodegradable, and non‐toxic food packaging (Chen et al. 2012). Studies have demonstrated its potential to improve food quality attributes, such as texture, appearance, and nutritional value, while adhering to food safety standards (Setsiba and Fawole 2022). However, *Aloe ferox* gel alone exhibits limited antimicrobial efficacy and preservation capacity (Viljoen et al. 2023). To address these limitations, incorporating raspberry pomace enhances its antimicrobial properties, antioxidant activity, and nutritional benefits, further strengthening its application in postharvest food quality management.

Raspberry pomace, a by‐product of the juice and jam industry, is a rich source of anthocyanins, polyphenols, and other bioactive compounds with potent antioxidant and antimicrobial properties (Yao et al. 2021). These compounds help inhibit spoilage‐causing microorganisms, extending shelf life and enhancing food safety (Tsegay and Mulaw 2024). While this study focuses specifically on raspberry waste, previous research has demonstrated similar biopreservative effects using by‐products from other fruits, including grape and clementine mandarin peels (Bambeni et al. 2021), as well as various Southern African indigenous fruits (Pfukwa et al. 2020), highlighting the broader potential of fruit‐derived waste in postharvest preservation strategies.

However, their instability under unfavorable conditions such as high temperature, light, oxygen, and pH during food processing and storage limits their direct application in foods (Yousefi et al. 2015). Encapsulation, a process in which bioactive compounds are enclosed in polymeric materials, enhances their stability, protects them from degradation, and facilitates their controlled release and integration into food systems (Makhathini and Fawole 2022). Encapsulated raspberry pomace (ERPP) improves the stability and functionality of its bioactives and enhances the antioxidant efficacy and performance of edible coatings in food applications (Nthimole et al. 2022).

Therefore, this study aims to evaluate the effect of AF (alone or in combination with ERPP) as a coating on ready‐to‐eat pomegranate arils during cold storage. *Aloe ferox* and this combination have not been extensively investigated, rendering our study one of the initial assessments of its effect on pomegranate arils.

## 2 Materials and Methods

### 2.1 Plant Material, Chemicals and Experimental Design

Freshly prepared pomegranate arils were procured from Ganico organic pomegranate farm in Johannesburg, Gauteng Province, South Africa (26°13′26.6″S, 28°04′47.8″E) and transported to the Postharvest and Agroprocessing Research Centre at the University of Johannesburg for treatment application and storage.

Red raspberry ( *Rubus idaeus* sp.) pomace, a by‐product of juice processing, was sourced from Berry Farm in Johannesburg. Upon collection, the pomace was dried at −55°C using a benchtop freeze‐dryer (Buchi Lyovapor L‐200, Germany). After drying, the material was ground into a fine powder using a Bennett Read Nutrition Extractor (1000 W) and sieved to obtain uniform particle sizes.

For extraction, the dried raspberry pomace powder was mixed with 70% ethanol and sonicated in a Labotec ultrasound bath (Cape Town, Western Cape, South Africa) to facilitate the extraction of bioactive compounds. The mixture was then filtered using Whatman filter paper No. 1, followed by centrifugation with a Thermo Fisher Scientific Biofuge centrifuge (Stratos, United Kingdom) at 5590 × g and 4°C for 5 min. The supernatant was concentrated to 9°Brix using a BUCHI Rotavapor R‐300 (Flawil, Switzerland) at 50°C under reduced pressure (150 mbar).

For encapsulation, the concentrated raspberry pomace extract was heated to 50°C on a hot plate and mixed with wall material ( *Opuntia ficus‐indica* mucilage). The mixture was homogenized using a Stuart SHM2 ultra‐homogenizer (Staffordshire, United Kingdom) at medium‐high speed for 2 min to ensure uniform dispersion. The homogenized samples were then frozen at –80°C for 24 h, followed by freeze‐drying using a BUCHI Lyovapor L‐200 freeze‐dryer (Flawil, Switzerland) at –55°C and 0.03 mbar for 72 h. The resulting encapsulated raspberry pomace powder was stored in airtight containers for further use.

In addition to the raspberry pomace, *Aloe ferox* gel was obtained from Organic Aloe (Pty) Ltd., based in Alberta, South Africa. The aloe gel was freeze‐dried under the same conditions (−55°C, 50 mTorr) for 36 h to preserve its bioactive compounds and improve storage stability. The freeze‐dried aloe gel was then pulverized into a fine powder before being used in subsequent experimental procedures. All the used chemicals were purchased from Sigma‐Aldrich Co. (Johannesburg, South Africa) and Merck Millipore Co. (Johannesburg, South Africa). The study followed a Randomized Complete Block Design (RCBD) with four treatments, each replicated three times.

### 2.2 Coating Preparation and Application

The AF‐based edible coating, enriched with encapsulated raspberry pomace powder (ERPP), was prepared by heating distilled water to 50°C. *Aloe ferox* (3 g) and cellulose nanofiber (CNF) (1%) were then added to the water and stirred magnetically for 30 min. Different concentrations of ERPP (0.3% and 0.6%) and glycerol (30%) were incorporated into the solution, followed by an additional 15 min of magnetic stirring. The mixture was subsequently homogenized for 30 s. The solution underwent sonication for 10 min using an ultrasonic bath (705, Labotec, Johannesburg, Gauteng, South Africa) to remove any bubbles.

The pomegranate arils were randomly divided into four treatment groups, each containing 2.5 kg of arils. These groups were immersed in their respective coating formulations for 5 min to ensure uniform coating application. The excess solution was drained using a strainer, and the arils were placed on sanitized trays to air dry at 23°C ± 2°C and 65% ± 5% relative humidity. Control samples were rinsed with distilled water. Once dry, the arils were packed in polypropylene punnets (100 g per punnet) and stored at 4.5°C ± 0.5°C with 85% ± 0.5% relative humidity for 18 days. Sampling was conducted in 3‐day intervals.

### 2.3 Weight Loss, Respiration Rate and Firmness

The weight loss of the stored arils was determined by randomly weighing four punnets for each treatment on each sample day using a high‐precision laboratory balance (Labotec Precision Balance, XP20K1, China; 0.001 g accuracy) (Mwelase et al. 2023).

The respiration rate was determined on each sampling day using the closed system method as described by Fawole and Opara (2013b) with slight modifications. 100 g in each treatment were incubated in triplicate in a 250 mL airtight container at ambient temperature for 2 h. An infrared gas analyzer (Dansensor CheckPoint 3) was used to monitor carbon dioxide gas generation within the container following incubation. The results were presented as mL·CO_2_/kg·h.

The firmness of arils was measured using the Fawole and Opara (2013c) method with an Agrosta texture meter (Agrosta texture analyzer) equipped with a 35 mm compression probe. The instrument's working settings were 1 mm/s probe speed and 0.30 N force. Texture was evaluated with 10 arils per treatment in triplicate. The highest force necessary to create this deformation was measured and presented in Newtons (N).

### 2.4 Color Attributes

The color of pomegranate arils was measured using a Minolta Chroma Meter Model CR‐400 (Minolta, Tokyo, Japan). The results were expressed as a mean value from three replications of the measured samples. The degree of browning was expressed as L* and a* values. Equations (1), (2) and (3) were used to calculate the hue angle (h°), chroma (C*), and total color differences (TCD), respectively.

(1) $$h^{{}^{\circ}}=\left(\frac{b*}{a*}\right)$$

(2) $$C*={\left(a{*}^{2}+b{*}^{2}\right)}^{\frac{1}{2}}$$

(3) $$\mathrm{\Delta E}=\sqrt{{\left(L^{*}o-L^{*}\;\right)}^{2}+{\left(a^{*}o-a^{*}\;\right)}^{2}+{\left(b^{*}o-b^{*}\;\right)}^{2}}$$

The FD sample's ($L_{0}^{*}$, $a_{0\;}^{*}\text{and}\;b_{0}^{*}$) and SD sample values (𝐿*, 𝑎*, and 𝑏*) were measured in triplicate for each treatment.

### 2.5 Determination of Titratable Acidity (TA) and Total Soluble Solids (TSS)

TSS was determined using a digital handheld refractometer (Atago Refractometer PAL‐1, Atago) calibrated to zero with double‐distilled water, and the results were expressed as ^o^Brix. TA content was determined using an Orion Star T940 All‐In‐One pH Titrator (ThermoFisher Scientific, Horsham, Sussex, UK). To prepare, 2 mL of crude pomegranate juice (PJ) was diluted with 90 mL of double‐distilled water and titrated to pH 8.2 using 0.1 M sodium hydroxide (NaOH). The data were presented as citric acid (%) equivalents. The results were presented as citric acid (%) equivalents.

### 2.6 Phytochemical Analysis

#### 2.6.1 Total Phenolic Content

The total phenolic content (TPC) in PJ methanolic extracts was determined using the Folin–Ciocalteu (Folin‐C) colourimetric method with slight modifications (Sari et al. 2023). A 1 mL sample of pomegranate juice was mixed with 10 mL of 50% methanol and vortexed for 30s to ensure thorough mixing. The mixture was then sonicated for 10 min and centrifuged at 10,000 × g for 10 min at 4°C. A 20 μL aliquot of clear supernatant was pipetted into a 96‐well microplate and mixed with an equivalent volume (20 μL) of Folin‐C reagent. After a 5‐min incubation, 200 μL of 7% sodium carbonate solution and 10 μL distilled water were added to each well. The reaction mixture was incubated for 40 min at room temperature in the dark. Absorbance was measured at 700 nm using a Microplate Reader (AMR‐100, China), with a blank well used as a reference. TPC was presented as milligrams of gallic acid equivalents per gram of dry weight (mg GAE/g DW).

#### 2.6.2 Total Anthocyanin Content

Total anthocyanin content (TAC) was quantified using the pH differentiation method described by Fawole and Opara (2013b). The pomegranate juice extract was combined with two different buffers, one at pH 1 (potassium chloride) and the other at pH 4.5 (sodium acetate). The absorbances (A) were determined for each buffer using a UV Visible Spectrophotometer (SP‐UV 300, Shanghai, China) at two different wavelengths (520 and 700 nm). Equations (4) and (5) were used to calculate TMA, and the results were expressed as mg C3gE/100 mL RBJ or 100 g RBJP, or milligram cyanidin‐3‐glucoside equivalent per 100 mL RBJ or 100 g RBJP.

(4) $$A={\left(A_{510}–A_{700}\right)}_{\mathrm{pH}\;1.0}+{\left(A_{510}–A_{700}\right)}_{\mathrm{pH}\;4.5}$$

(5) $$\text{Total anthocyanin content}\;\left(\mathrm{mg}/\mathrm{mL}\right)=A\times \mathrm{MW}\times \mathrm{DF}/\varepsilon \times L$$

A, absorbance value; Є, cyanidin‐3‐glucoside molar absorbance ~26,900; MW, cyanidin‐3‐glucoside molecular weight (449.2 g/mol); DF, dilution factor; L, cell path length ~1 cm; and TAC, total anthocyanin content.

### 2.7 Antioxidant Activity

#### 2.7.1 DPPH Radical Scavenging Activity

The DPPH radical‐scavenging activity was measured using a method described by Jurić et al. (2023). 15 μL of the pomegranate juice methanolic extract was mixed with 235 μL of methanolic 2,2‐diphenyl‐1‐picryl‐hydrazine (DPPH) solution (0.1 mM) and incubated in the dark for 30 min before reading absorbance at 520 nm. The antioxidant activity was presented as a percentage (%), as shown in Equation (6).

(6) $$\text{Radical scavenging activity}\;\left(\%\right)=\frac{A_{\text{control}}-A_{\text{sample}}}{A_{\text{control}}}\times 100$$

A, Absorbance value.

#### 2.7.2 Ferric Reducing Antioxidant Power (FRAP) Assay

FRAP was quantified using a method described by Marapana and Maduwanthi (2021). The FRAP reagent (150 μL) and acetate buffer (30 μL) were added to 20 μL of methanolic pomegranate aril juice extract sample. The combination was allowed to react in the dark for 30 min before measuring the absorbance of FRAP at 590 nm. The data were expressed as mg TE/g, as calculated from the Trolox calibration curve.

### 2.8 Statistical Analysis

An unbalanced design was analyzed using Genstat 20th Edition, VSN International, Hemel Hempstead, UK20^th^ Ed. Mean values were separated by the Bonferroni test using the least significant difference (LSD) test at the 5% level of significance. The graphs and tables were prepared with Microsoft Excel. Principal component analysis (PCA) was carried out using the statistical software XLSTAT Version 2020.4.1.1020 (Addinsoft, Paris, France).

## 3 Results and Discussion

### 3.1 Weight Loss

**FIGURE 1:**
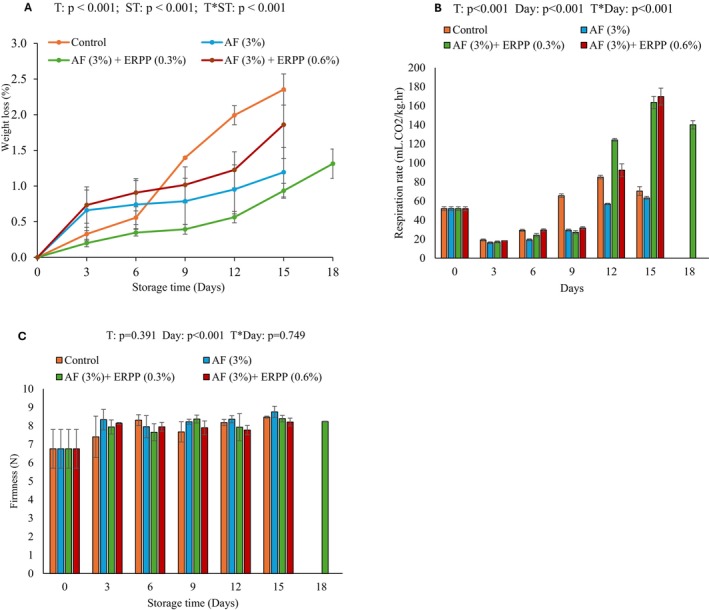
Effect of AF‐based edible coating enriched with ERPP on (A) Weight loss, (B) Respiration rate, and (C) Firmness of ‘ready‐to‐eat’ pomegranate arils during storage at 4.5°C for 18 days. After day 15 control, AF only, and AF + ERRP (0.6%) were terminated. AF, *Aloe ferox* ; ERPP, encapsulated raspberry pomace powder; ST, storage time; T, treatment; T*ST, interaction between treatment and storage time. Values are presented as means ± SE from three replicates (*n* = 3), where error bars represent SEM.

Figure 1A presents the cumulative weight loss of pomegranate arils over 15 days of cold storage, highlighting the effects of coating treatments. The treatment and storage duration interaction significantly influenced weight loss (*p* < 0.001). Weight loss increased in both treated and untreated samples, with an average of 2.35% in control, 1.19% (AF), 0.93% (AF + 0.3% ERPP) and 1.86% (AF + 0.6% ERPP). While both treated and untreated arils showed increasing weight loss over time, treated arils exhibited a slower loss rate, indicating the coatings' effectiveness. Among the treatments, the combination of AF and ERPP at 0.3% was most effective in reducing weight loss, outperforming AF alone and AF incorporated with 0.6% ERPP. This suggests ERPP enhances weight retention, but higher concentrations may reduce efficacy. The AF + 0.3% ERPP coating extended the shelf life of “ready‐to‐eat” pomegranate arils by 3 days compared to other treatments. Research on the application of *Aloe ferox* (AF) for controlling weight loss in fresh produce remains limited. However, Setsiba and Fawole (2022) demonstrated that AF gel effectively delayed weight loss in ‘Kinnow’ mandarins during cold storage. Additionally, Setsiba and Fawole (2022) also discovered that it controlled water vapor permeability, which is directly associated with weight reduction since it influences the pace at which water vapor evaporates from the fruit (Jafarzadeh et al. 2021). This effect is attributed to the polysaccharide composition of AF, which regulates gaseous exchange and minimizes water loss, thereby preserving the overall quality of fresh produce (Cruz‐Monterrosa et al. 2023; Comas‐Serra et al. 2023). These findings align with studies on polysaccharide‐based coatings, which underscore the efficacy of biopolymers in reducing weight loss and maintaining the quality of minimally processed products (Pan et al. 2024). In this study, the incorporation of ERPP into AF‐based coatings further enhanced their effectiveness, likely due to the antioxidant properties of ERPP polyphenols. The combined use of AF and ERPP creates a more robust barrier, effectively reducing moisture loss and preserving aril quality during storage.

### 3.2 Respiration Rate

The respiration rates of treated and untreated pomegranate arils during an 18‐day cold storage period are presented in Figure 1B. Statistical analysis revealed a significant interaction between treatment and storage duration (T* Day, *p* < 0.001). On day 0, all treatments exhibited elevated respiration rates (51.99 mL.CO_2_/kg.h), likely due to physiological stress from processing and handling (Waghmare et al. 2013). By day 3, respiration rates markedly decreased across treatments (19.09–16.16 mL.CO_2_/kg.h), attributed to the cold storage conditions that suppress metabolic activity by slowing enzymatic and respiratory processes (Aindongo et al. 2014). The respiration rate consistently increased with storage time; however, treated arils exhibited a delayed increase compared to control arils between day 3 and day 9. Treatments with AF alone significantly reduced respiration rates compared to those incorporated with ERPP at 0.3% or 0.6%. The increased respiration observed in the AF + ERPP treatments may result from polyphenolic compounds in ERPP enhancing metabolic activity (Lopez‐Corona et al. 2022). Despite this, the AF + ERPP (0.3%) treatment extended the shelf life by 3 days compared to AF alone, suggesting that elevated respiration may reflect a physiological adaptation rather than spoilage. This shows that this treatment contains polysaccharides and phenolic compounds, which are likely to regulate respiration rate, minimize gas exchange, slow down metabolic activities, and preserve fruit quality (Dhall 2013). The findings of this study are consistent with those reported by Caleb et al. (2012) for ‘Acco’ pomegranate arils, where edible coatings effectively delayed the respiration rate of treated fruits compared to untreated ones. In the present study, the reduced respiration rate observed in treated arils can be attributed to the coatings' creation of a modified atmosphere. This altered atmosphere slowed the respiration process, which probably decreased the amount of oxygen in the treated fruits' internal environment (Prasanna et al. 2007). Furthermore, the fruit's physicochemical and phytochemical characteristics may be preserved by lowering metabolic activity and conserving energy reserves due to the delayed respiration rate. This mechanism demonstrates how the edible coatings influence the microenvironment surrounding the fruit while also acting as a physical barrier to gas exchange and moisture loss (Hira et al. 2022). The changed atmosphere formed by the coatings contributes to the retention of quality qualities, such as color stability, texture, and antioxidant properties, coinciding with consumer desires for fresh, minimally processed fruits. Future studies should investigate the tuning of coating (ERPP concentrations) formulations to enhance these benefits and explore their applicability across a larger spectrum of fresh food.

### 3.3 Firmness

Fruit firmness is a standard quality indicator that consumers observe when purchasing fruits (Rahman et al. 2021); therefore, this study explored the impact of using AF‐based edible coatings on the firmness of pomegranate arils (Figure 1C). The results showed minimal changes in fruit firmness throughout the storage period; by day 15, no significant differences in firmness were detected among treatments. This suggests that pomegranate arils maintain their firmness well during cold storage due to their intrinsic structural integrity (Leta et al. 2024). These findings align with previous studies by Caleb et al. (2013) and Ayhan and Eştürk (2009), who reported little to no firmness changes in ‘Acco’, ‘Herskawitz’, and ‘Hicaznar’ arils during 14 days of cold storage. The breakdown of insoluble protopectin into soluble pectin or enhanced membrane permeability brought on by cell division is the two main causes of fruit softening, which often occurs as fruit ages and ripens (Kaur and Kaur 2019). Water loss speeds up changes in membrane permeability, which causes fruits to lose water more quickly when stored in cold temperatures (Bartz and Brecht 2002). However, in this study, it was observed that coating pomegranate arils with AF + 0.3% ERPP further managed to extend the shelf life of the pomegranate arils by an extra 3 days. *Aloe ferox* (AF) contains several chemical components that assist in maintaining fruit firmness when used in edible coatings; for example, polysaccharides like acemannan and glucomannan (Comas‐Serra et al. 2023). These polysaccharides establish a barrier that inhibits water loss and gas exchange, slowing down the rate of respiration and ethylene generation in fruits. This helps maintain firmness by inhibiting the enzymatic breakdown of cell walls (Dhall 2013). Furthermore, AF and ERPP contain phenolic compounds such as anthraquinones, phenolic acids, and aloin, saponins with antioxidant properties that reduce oxidative stress in fruits, as well as antimicrobial properties that help prevent microbial spoilage, thereby protecting cell wall integrity and delaying senescence (Shala et al. 2024).

### 3.4 Color Attributes

Table 1 shows the changes in redness (a*), hue, and chroma of treated and untreated “ready‐to‐eat” pomegranate arils during the 18‐day storage period. Both treated and untreated arils showed an initial increase in redness, followed by a gradual decline over time, with a significant interaction between treatment and storage period. Treated arils exhibited greater redness than the untreated ones, with those coated with 0.3% AF + ERPP maintaining the highest a* values with an average of 21.07, followed by AF + ERPP (0.6%) and AF alone with 16.37. The incorporation of ERPP proved effective in preserving the color, deepening the shade from pink to rose, similar to the findings of Yang et al. (2021). Additionally, hue angle decreased significantly (*p* < 0.05) in both groups, with untreated and AF + ERPP (0.6%) samples showing a more noticeable increase, indicating greater color degradation. Chroma values slightly increased throughout storage, with treated arils showing significantly higher values compared to controls. This shows that AF incorporated with ERPP and AF alone were more efficient in preserving the color of pomegranate arils during storage, a key quality attribute that aligns with consumer preference for visually appealing.

**TABLE 1:** Color attributes of “ready‐to‐eat” pomegranate arils treated with AF and AF incorporated with ERPP were evaluated during storage at 4.5°C over 18 days.

Parameter	Treatment	Storage time (days)						
		0	3	6	9	12	15	18
Redness (a*)	Control	13.4 ± 1.70^a^	18.97 ± 1.46^ab^	16.27 ± 3.26^a^	16.07 ± 1.65^a^	16.97 ± 2.78^a^	15.67 ± 1.00^ab^	
	AF (3%)	13.4 ± 1.70^a^	16.97 ± 1.01^b^	18.47 ± 3.78^a^	12.73 ± 2.54^a^	15.90 ± 4.46^a^	16.37 ± 2.49^ab^	
	AF (3%) + ERPP (0.3%)	13.4 ± 1.70^a^	23.57 ± 2.47^a^	18.47 ± 2.48^a^	17.23 ± 1.65^a^	17.30 ± 1.68^a^	21.07 ± 1.89^a^	19.03 ± 1.70
	AF (3%) + ERPP (0.6%)	13.4 ± 1.70^a^	20.27 ± 1.88^ab^	19.37 ± 2.95^a^	17.60 ± 1.42^a^	18.57 ± 4.67^a^	12.07 ± 2.34^b^	
Significant level (*p*)	Treatment (A) 0.004	Storage Time (B) < 0.001		A × B 0.043				
Chroma (C*)	Control	17.01 ± 2.00^a^	20.30 ± 1.12^a^	19.36 ± 0.29^a^	19.96 ± 0.55^a^	21.01 ± 0.17^a^	19.26 ± 0.05^ab^	
	AF (3%)	17.01 ± 2.00^a^	21.45 ± 0.56^a^	21.68 ± 0.60^a^	16.14 ± 0.14^a^	19.27 ± 0.19^a^	19.96 ± 0.30^ab^	
	AF (3%) + ERPP (0.3%)	17.01 ± 2.00^a^	26.87 ± 0.38^a^	22.80 ± 0.46^a^	20.21 ± 0.21^a^	21.16 ± 0.73^a^	24.39 ± 0.18^a^	16.93 ± 2.83
	AF (3%) + ERPP (0.6%)	17.01 ± 2.00^a^	23.13 ± 0.05^a^	21.95 ± 0.24^a^	20.36 ± 0.37^a^	21.52 ± 0.24^a^	16.24 ± 0.21^b^	
Significant level (*p*)	Treatment (A) 0.242	Storage Time (B) < 0.001		A × B 0.166				
Hue angle (*h*°)	Control	1.24 ± 0.15^a^	0.41 ± 0.52^a^	0.99 ± 0.17^a^	1.15 ± 0.25^a^	1.15 ± 0.17^a^	1.11 ± 0.14^a^	
	AF (3%)	1.24 ± 0.15^a^	1.21 ± 0.12^a^	0.97 ± 0.18^a^	1.23 ± 0.13^a^	1.09 ± 0.30^a^	1.09 ± 0.16^a^	
	AF (3%) + ERPP (0.3%)	1.24 ± 0.15^a^	0.85 ± 0.05^a^	1.14 ± 0.35^a^	0.96 ± 0.07^a^	1.10 ± 0.14^a^	0.91 ± 0.13^a^	22.26 ± 0.02
	AF (3%) + ERPP (0.6%)	1.24 ± 0.15^a^	0.85 ± 0.21^a^	0.81 ± 0.45^a^	0.91 ± 0.05^a^	0.93 ± 0.11^a^	1.42 ± 0.29^a^	
Significant level (*p*)	Treatment (A) 0.229	Storage time (B) 0.001		A × B 0.010				

*Note:* After day 15 control, AF only and AF + ERRP (0.6%) were terminated. Values are presented as means ± SE from three replicates (*n* = 3).

Abbreviations: AF, *Aloe ferox*; ERPP, encapsulated raspberry pomace powder; ST, storage time; T, treatment; T*ST, interaction between treatment and storage time. Bonferroni test indicates that values in columns, for each attribute, with different superscripts (“a” and “b”) are significantly different (*p* < 0.05).

### 3.5 Titratable Acidity and Total Soluble Solids

**FIGURE 2:**
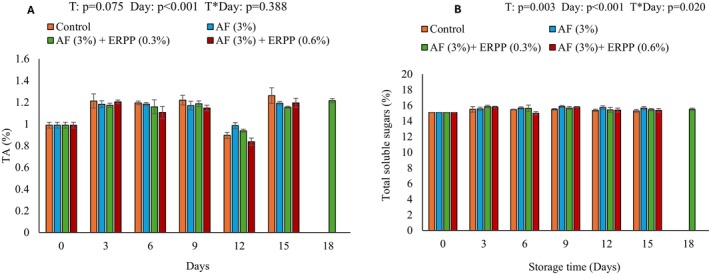
Effect of AF‐based edible coating enriched with ERPP on (A) TA and (B) TSS of ‘ready‐to‐eat’ pomegranate arils during storage at 4.5°C for 18 days. After day 15 control, AF only, and AF + ERRP (0.6%) were terminated. AF, *Aloe ferox* ; ERPP, encapsulated raspberry pomace powder; ST, storage time; T, treatment; T*ST; interaction between treatment and storage time; TA, titratable acidity; TSS, total soluble solids. Values are presented as means ± SE from three replicates (*n* = 3), where error bars represent SEM.

Titratable acidity is a crucial quality parameter that significantly influences both the flavor profile and preservation of perishable products, as it reflects the organic acid content that contributes to tartness and microbial stability (Holland et al. 2009). Figure 2A illustrates the variations in titratable acidity (TA) observed in treated and untreated pomegranate arils over 18 days of cold storage. The data reveal distinct trends in the TA levels between treated and untreated samples, highlighting the efficacy of different preservation strategies. A slight increase was observed in both treated and untreated pomegranate arils during the early days of storage, increasing from 0.99% to 1.21%. Pomegranate arils were more effective at sustaining TA after treatment (AF alone and 0.3% ERPP). This stability could be explained by the applied treatments' inhibitory effects on metabolic processes, which slow down organic acid degradation (Thapa et al. 2020). Preservation methods, such as edible coatings or natural antimicrobial agents, could play a role in maintaining the acid content and extending the shelf life of the arils. However, on day 12 there was a decline across all treatments, possibly due to metabolic activities such as respiration and the conversion of organic acids into simpler molecules. A rapid decrease in TA is associated with faster senescence, and therefore edible coatings as surface barriers modify the internal atmosphere of the fruit to prevent the reduction in TA content through acid metabolism (Guroo et al. 2021). This reduction in acidity can compromise flavor quality, leading to a sweeter and less tart profile, as well as increased susceptibility to microbial spoilage. These results corroborate with Kawhena et al. (2020), who observed an increase in TA for pomegranate arils coated with gum Arabic and methyl cellulose coatings enriched with thyme oil after 12 days of cold storage (5°C). Furthermore, AF + ERPP (0.3%) maintained TA until day 18, which shows that the edible coatings of polyphenols and polysaccharides maintained TA during storage. This may result from a delayed respiratory process that could have reduced the utilization of organic acids during storage (Kaur et al. 2023). Changes in TA alter the taste of the arils, making them sour, which can be desirable or unfavorable depending on consumer preference. Maintaining optimal TA levels is essential for ensuring the overall acceptability and safety of minimally processed fruits like pomegranate arils during extended storage.

The total soluble solids (TSS) content of pomegranate arils was significantly influenced by both treatment and storage duration (Figure 2B). Over the storage period, a slight increase in TSS was observed, which can be attributed to the hydrolysis of starch into sugars, a desirable characteristic in pomegranate juice as it enhances sweetness and flavor (Das et al. 2013). The increase in TSS across treated and untreated pomegranate arils ranged from 15.1% to 15.9%, with all treatments successfully maintaining TSS levels throughout storage. Additionally, AF + ERPP (0.3%) maintained TSS until day 18, which shows that the combination of polysaccharides and polyphenols played a significant role in preserving TSS. According to González‐Aguilar et al. (2019), the stability of TSS indicates that the applied coating acted as an effective barrier, preventing significant moisture loss or degradation processes, such as enzymatic activity and respiration. Similarly, Oms‐Oliu et al. (2008) highlighted that such coatings minimize physiological changes in fruit during storage, thus preserving its chemical integrity. The maintained TSS levels suggest that the coatings not only retained the sweetness and flavor profile of the arils but also contributed to maintaining their overall quality and freshness (Kumar and Deb 2019). By reducing moisture loss and mitigating metabolic changes, the coatings likely enhanced the shelf life and marketability of the fruit, demonstrating their potential for extending the postharvest quality of fresh‐cut fruit (Maringgal et al. 2020). This highlights the effectiveness of edible coatings as a postharvest treatment for maintaining minimally processed fruits' sensory and nutritional properties.

### 3.6 Total Phenolic Content and Total Anthocyanin Content

**FIGURE 3:**
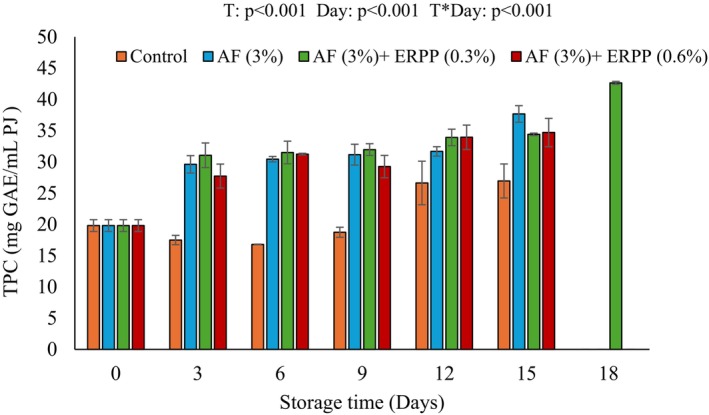
Effect of AF‐based edible coating enriched with ERPP on TPC of ‘ready‐to‐eat’ pomegranate arils during storage at 4.5°C for 18 days. After day 15 (control, AF only, and AF + ERRP (0.6%)) were terminated. AF, *Aloe ferox* ; ERPP, encapsulated raspberry pomace powder; ST, storage time; T, treatment; T*ST, interaction between treatment and storage time; TPC, total phenolic content. Values are presented as means ± SE from three replicates (*n* = 3), where error bars represent SEM.

Figure 3 shows the changes in total phenolic content (TPC) of pomegranate arils after storage. The interaction between storage duration and treatment significantly affected phenolic content. The increase in the storage time resulted in a significant rise in TPC (19.81 to 42.65 mg GAE/mL PJ), notably in treated arils compared to controls, particularly those coated with AF + ERPP (0.3%), showing an extended shelf life by an additional 3 days. This increase is affiliated with Mwelase et al. (2023) findings, which also reported that the phenolic content of pomegranate arils increased over time during preservation. This increase most likely results from enzymatic or non‐enzymatic browning events during storage, which liberate bound phenolics or generate new phenolic compounds (Martínez‐Hernández et al. 2021). The increase in phenolic content in treated arils is presumably due to the inclusion of ERPP in the coating formulations. ERPP is recognized for its elevated polyphenolic content, which may have facilitated the enhancement of the TPC in the arils, hence augmenting their total antioxidant potential. This effect is substantial, as phenolic chemicals are essential in the defense mechanisms of fresh produce, safeguarding cellular structures from oxidative damage during storage (Cisneros‐Zevallos and Jacobo‐Velázquez 2020; Hu et al. 2022). The potential of ERPP‐enriched coatings to increase phenolic content implies that such treatments might extend the shelf life and improve the nutritional value of minimally treated fresh fruit.

**FIGURE 4:**
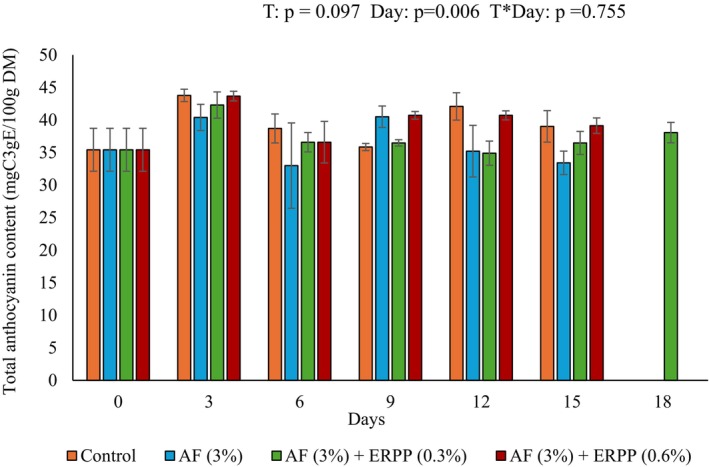
Effect of AF‐based edible coating enriched with ERPP on TAC of ‘ready‐to‐eat’ pomegranate arils during storage at 4.5°C for 18 days. After day 15 (control, AF only and AF + ERRP (0.6%)) was terminated. After day 15 (control, AF only and AF + ERRP (0.6%)) was terminated. AF, *Aloe ferox* ; ERPP, encapsulated raspberry pomace powder; ST, storage time; T, treatment; T*ST; interaction between treatment and storage time; TAC, total anthocyanin content. Values are presented as means ± SE from three replicates (*n* = 3), where error bars represent SEM.

Figure 4 shows the anthocyanins of treated and untreated pomegranate arils during storage. Anthocyanins are water‐soluble polyphenolic chemicals responsible for the red coloration of pomegranate arils (Arendse et al. 2014). The obtained result demonstrated that the total anthocyanin content in all treatments increased at day 3 of storage. The treatments generally maintained the levels of total anthocyanins with only a slight increase after 3 days. By the end of the day 15 storage period, arils with high anthocyanin values (39.13 mgC3gE/100 g DM) were those that were coated with AF+ ERPP (0.6%) followed by Control (39.03 mgC3gE/100 g DM), AF+ ERPP (0.3%) (36.49 mgC3gE/100 g DM) and AF only (33.42 mgC3gE/100 g DM). However, after 15 days of storage, treatments AF+ ERPP (0.6%), Control, and AF only were terminated due to the deterioration in quality. Treatment AF+ ERPP (0.3%) maintained its quality up to day 18, suggesting the efficacy of the treatment. This is likely due to the polyphenolic compounds that are found in both *aloe ferox* and raspberry that act as antioxidants, forming a protective coating around the arils and reducing oxidative stress, which helps prevent the degradation of anthocyanins (Shahidi and Ambigaipalan 2015). High TAC is typically connected with improved color quality and more antioxidant activity, and anthocyanins enhance the fruit's visual attractiveness and health advantages (Ozgen et al. 2008).

### 3.7 DPPH Radical‐Scavenging Activity and Ferric‐Reducing Antioxidant Power (FRAP) Assays

**FIGURE 5:**
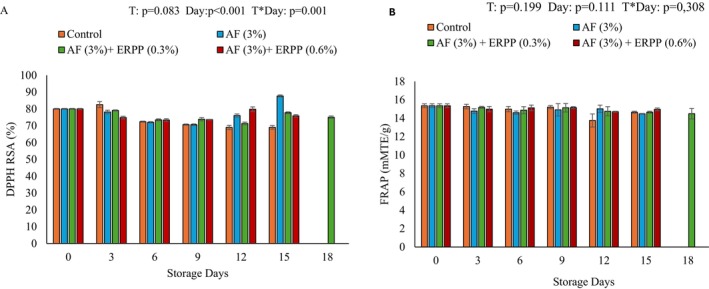
Effect of AF‐based edible coating enriched with ERPP on RSA (A) and FRAP (B) of ‘ready‐to‐eat’ pomegranate arils during storage at 4.5°C for 18 days. After day 15 (control, AF only, and AF + ERRP (0.6%)) were terminated. AF, *Aloe ferox* ; ERPP, encapsulated raspberry pomace powder; RSA, radical scavenging activity; ST, storage time; T, treatment; T*ST, interaction between treatment and storage time. Values are presented as means ± SE from three replicates (*n* = 3), where error bars represent SEM.

Figure 5A shows the effects of coating treatments and storage times on DPPH radical‐scavenging activity (RSA). The radical‐scavenging activity of pomegranate arils declined gradually with storage in all the treatments. However, the treated pomegranate arils had the highest values of radical scavenging activity, AF alone with 87.64%, AF with 0.3% ERPP (77.73%) with an extended 3 days (75.07%), AF + ERPP (0.6%) (75.86%) and control with 69.04% on the last day of storage. This study's results align with those reported by Mwelase et al. (2022), who observed a similar decrease in the radical scavenging activity of the minimally processed pomegranate arils with the storage time, regardless of treatment. However, the 0% ERPP and 0.6% ERPP showed an increase on day 12, whereas 0.3% ERPP showed a slight increase on day 15. The control arils exhibited a rapid decline in RSA, whereas the coated arils maintained RSA more effectively. This suggests that polyphenols may function as bridging agents, enhancing the binding affinity between the components and reducing their interactions (Chollakup et al. 2020). Also, there was a decrease in FRAP in all treatments, but there was no significant difference between the treatment and the storage time (Figure 5B). Rapid decline was observed in control at day 12 (from 15.36 to 13.75 mMTE/mL PJ), while the coated arils showed significantly better retention of FRAP (ranging between 14.70 and 15.02 mMTE/g) and continued retaining it till the final day of storage. This shows that the antioxidant capacity of the arils is either improving or remaining stable over the storage period. This means that the arils are stable and not degrading over the storage period, and also helps preserve the quality and health benefits of the arils. The delocalization of electrons within the benzene ring structure of polyphenols may be the mechanism by which polyphenols improve the antioxidant performance of composite materials (Pan et al. 2024). When polyphenols interact with polysaccharides, they encourage the ionization of phenolic hydroxyl groups, which increases the coatings' capacity to donate hydrogen, thus improving their antioxidant activity. Additionally, the antioxidant capability of polysaccharide‐polyphenol composites results from their capacity to scavenge free radicals, nitrogen oxides, hydrogen peroxide, and chelate iron ions (Pan et al. 2024).

### 3.8 Principal Component Analysis and Correlation Matrix

#### 3.8.1 Principal Component Analysis

**FIGURE 6:**
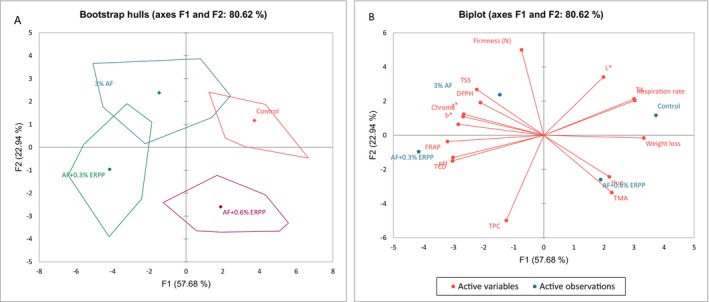
Principal component analysis (PCA) bootstrap hulls (A), PCA biplot (B) showing the correlation between the measured parameters and coating treatment during the last day (day 15) of storage.

Figures 6A,B present principal component analysis (PCA) biplots and bootstrap hull plots, respectively, which were used to differentiate the coating treatments based on parameters assessed on the final storage day. The PCA identified two principal components (F1 and F2), which explained 80.62% of the data variability. The first component (F1) accounted for 57.68% of the variance, while the second component (F2) explained 22.94%. The bootstrap hull plot identified six groups: the control, 3% AF, AF + ERPP (0.3%), and AF + ERPP (0.6%).

The PCA biplot revealed the features driving these groupings. The first group, consisting of AF alone, was characterized by high firmness, total soluble solids (TSS), DPPH, a*, Chroma, b*, and L* values, indicating that this coating effectively reduced moisture loss and oxidative degradation, preserving the structural integrity, sugar content, antioxidants, and color of the arils. In contrast, the control group showed higher levels of weight loss, respiration rate, titratable acidity (TA), and low L* values, suggesting a loss in aril quality. The AF + ERPP (0.6%) group exhibited high total anthocyanin content (TAC) and hue, suggesting the preservation of anthocyanins, though there was a decline in color quality. Lastly, the AF + ERPP (0.3%) group displayed elevated FRAP, total phenolic content (TPC) and pH, indicating that this treatment effectively preserved and enhanced the arils' antioxidant capacity, polyphenolic stability, and overall quality during storage.

These findings suggest that both AF alone and AF combined with ERPP (0.3%) are suitable for food applications, as they effectively maintain moisture, reduce oxidative stress, and preserve color, texture, and antioxidant properties in pomegranate arils.

#### 3.8.2 Correlation Matrix

Table 2 presents the correlations between the evaluated parameters on day 18 of storage. A strong negative correlation was observed between the redness of pomegranate arils and hue (*r* = −0.964), indicating that an increase in the redness of the arils corresponds to a decrease in the hue angle. This suggests that the delay in fruit ripening was associated with higher redness. Weight loss exhibited a weak negative correlation with firmness (*r* = −0.293), indicating that greater weight loss was associated with reduced firmness, a sign of senescence. Additionally, weight loss showed a strong positive correlation with the respiration rate (*r* = 0.911), suggesting that increased weight loss was accompanied by an elevated respiration rate. The higher respiration rate likely accelerates the breakdown of stored carbohydrates, contributing to rapid weight loss. Titratable acidity (TA) demonstrated a strong negative correlation with total phenolic content (TPC) (*r* = −0.693), DPPH (*r* = −0.572), and FRAP (*r* = −0.796). These negative associations suggest that higher acidity in pomegranate arils is linked to lower levels of phenolic compounds and antioxidant activity. Conversely, a decrease in TA may indicate an increased presence of phenolic compounds, which are associated with enhanced antioxidant capacity, as assessed by DPPH and FRAP (Mwelase and Fawole 2022).

**TABLE 2:** Pearson's correlation matrix between quality attributes of uncoated (control) and coated pomegranate arils stored for 18 days at 4.5°C plus 3 days at 4.5°C.

Variables	a*	Hue	Chroma	Weight loss	Respiration rate	Firmness (N)	TSS	TA	TPC	TAC	DPPH	FRAP
a*	**1**											
Hue	**−0.964**	**1**										
Chroma	**1.000**	**−0.957**	**1**									
Weight loss	−0.681	0.540	−0.686	**1**								
Respiration rate	−0.485	0.262	−0.499	0.911	**1**							
Firmness (N)	0.224	−0.388	0.200	−0.293	0.093	**1**						
TSS	0.313	−0.307	0.302	−0.761	−0.529	0.773	**1**					
TA	−0.475	0.250	−0.490	0.903	**1.000**	0.110	−0.515	**1**				
TPC	0.083	0.175	0.110	−0.333	−0.680	−0.790	−0.231	−0.693	**1**			
TAC	−0.441	0.467	−0.427	0.747	0.454	−0.845	**−0.980**	0.439	0.340	**1**		
DPPH	0.176	−0.136	0.168	−0.746	−0.583	0.669	**0.980**	−0.572	−0.112	−0.920	**1**	
FRAP	0.909	−0.773	0.917	−0.876	−0.802	0.054	0.414	−0.796	0.416	−0.466	0.349	**1**

*Note:* Values in bold are different from 0 with a significance level of alpha = 0.05.

Abbreviations: a*, redness; DPPH, 2,2‐diphenyl‐1‐picryl hydrazyl; FRAP, free radical scavenging activity, and ferric ion reducing antioxidant power; TA, titratable acidity; TAC, total anthocyanin content; TPC, total phenolic content; TSS, total soluble solids.

## 4 Conclusion

This study shows the efficiency of *Aloe ferox* (AF)‐based edible coatings, notably when paired with encapsulated raspberry pomace powder (ERPP) at 0.3%, in retaining the quality of ready‐to‐eat pomegranate arils. The AF alone and AF + 0.3% ERPP coating efficiently maintained the physiological, physicochemical, chemical, phytochemical, and antioxidant qualities of the arils, increasing their shelf life while minimizing weight loss and retaining firmness. The findings highlight the potential of plant‐based, edible coatings as sustainable and functional solutions for postharvest fruit preservation. AF coating with 0.3% ERPP was particularly effective in preserving the pomegranate aril quality and shelf life, outperforming higher ERPP concentrations and untreated controls. The results also emphasize the importance of optimizing functional ingredient concentrations to maximize the benefits of such coatings. The findings of this study hold significant relevance for the food industry, particularly in advancing the development of minimally processed, ready‐to‐eat fruit products. Coatings formulated from AF and enriched with ERPP demonstrated potential to extend the shelf life of fresh‐cut fruits, thereby reducing postharvest losses and enhancing consumer appeal. The incorporation of ERPP enhances the antioxidant properties of the coating, addressing the increasing consumer demand for health‐conscious and functional food products. Moreover, the use of plant‐based, biodegradable materials such as Aloe ferox supports environmental sustainability by minimizing reliance on synthetic preservatives and packaging, contributing to more ecologically responsible food manufacturing practices.

## Author Contributions

**N. P. Mbonambi:** data curation (lead), investigation (lead), methodology (lead), writing – original draft (lead). **F. Seke:** project administration (equal), supervision (equal), writing – review and editing (equal). **S. Mwelase:** validation (equal), visualization (equal), writing – review and editing (equal). **O. A. Fawole:** conceptualization (lead), funding acquisition (lead), project administration (lead), supervision (lead), writing – review and editing (lead).

## Conflicts of Interest

The authors declare no conflicts of interest.

## Data Availability

The data that support the findings of this study are available on request from the corresponding author. The data are not publicly available due to privacy or ethical restrictions.
